# The Genome of the Mimosoid Legume *Prosopis cineraria*, a Desert Tree

**DOI:** 10.3390/ijms23158503

**Published:** 2022-07-31

**Authors:** Naganeeswaran Sudalaimuthuasari, Rashid Ali, Martin Kottackal, Mohammed Rafi, Mariam Al Nuaimi, Biduth Kundu, Raja Saeed Al-Maskari, Xuewen Wang, Ajay Kumar Mishra, Jithin Balan, Srinivasa R. Chaluvadi, Fatima Al Ansari, Jeffrey L. Bennetzen, Michael D. Purugganan, Khaled M. Hazzouri, Khaled M. A. Amiri

**Affiliations:** 1 Khalifa Center for Genetic Engineering and Biotechnology, United Arab Emirates University, Al Ain P.O. Box. 15551, United Arab Emirates; naganeeswaran@uaeu.ac.ae (N.S.); Rashid.ali@uconn.edu (R.A.); martin@uaeu.ac.ae (M.K.); rafi.m@uaeu.ac.ae (M.R.); alnuaimi_m@uaeu.ac.ae (M.A.N.); ajaymishra24@uaeu.ac.ae (A.K.M.); jithinb@uaeu.ac.ae (J.B.); 2 Mitrix Bio., 400 Farmington Ave., Farmington, CT 06032, USA; 3 Department of Biology, College of Science, United Arab Emirates University, Al Ain P.O. Box. 15551, United Arab Emirates; biduth.k@uaeu.ac.ae (B.K.); r.almeskari@uaeu.ac.ae (R.S.A.-M.); f.alansari@uaeu.ac.ae (F.A.A.); 4 Department of Genetics, University of Georgia, Athens, GA 30602, USA; xwwang@uga.edu (X.W.); src@uga.edu (S.R.C.); maize@uga.edu (J.L.B.); 5 Center for Genomics and Systems Biology, New York University Abu Dhabi, Abu Dhabi P.O. Box. 129188, United Arab Emirates; mp132@nyu.edu; 6 Center for Genomics and Systems Biology, New York University, New York, NY 10003, USA

**Keywords:** abiotic stress response genes, mesquites, NBS-LRR gene amplification, retrogenes, terpenoid synthesis genes

## Abstract

The mimosoid legumes are a clade of ~40 genera in the Caesalpinioideae subfamily of the Fabaceae that grow in tropical and subtropical regions. Unlike the better studied Papilionoideae, there are few genomic resources within this legume group. The tree *Prosopis cineraria* is native to the Near East and Indian subcontinent, where it thrives in very hot desert environments. To develop a tool to better understand desert plant adaptation mechanisms, we sequenced the *P. cineraria* genome to near-chromosomal assembly, with a total sequence length of ~691 Mb. We predicted 77,579 gene models (76,554 CDS, 361 rRNAs and 664 tRNAs) from the assembled genome, among them 55,325 (~72%) protein-coding genes that were functionally annotated. This genome was found to consist of over 58% repeat sequences, primarily long terminal repeats (LTR-)-retrotransposons. We find an expansion of terpenoid metabolism genes in *P. cineraria* and its relative *Prosopis alba*, but not in other legumes. We also observed an amplification of NBS-LRR disease-resistance genes correlated with LTR-associated retrotransposition, and identified 410 retrogenes with an active burst of chimeric retrogene creation that approximately occurred at the same time of divergence of *P. cineraria* from a common lineage with *P. alba*~23 Mya. These retrogenes include many biotic defense responses and abiotic stress stimulus responses, as well as the early Nodulin 93 gene. Nodulin 93 gene amplification is consistent with an adaptive response of the species to the low nitrogen in arid desert soil. Consistent with these results, our differentially expressed genes show a tissue specific expression of isoprenoid pathways in shoots, but not in roots, as well as important genes involved in abiotic salt stress in both tissues. Overall, the genome sequence of *P. cineraria* enriches our understanding of the genomic mechanisms of its disease resistance and abiotic stress tolerance. Thus, it is a very important step in crop and legume improvement.

## 1. Introduction

Legumes (Fabaceae) are a key flowering plant family, associated with the unique ability to fix nitrogen in soil though interaction with microbes. Traditionally, Fabaceae is classified in three subfamilies, Papilionoideae, Mimosoideae, and Caesalpinioideae [1]. A molecular phylogenetic analysis suggests a paraphyletic Caesalpinioideae, with the Mimosoideae forming a clade within the Caesalpinioideae [2]. The time between the origin and the major diversification of Fabaceae was estimated to be around 1–2.5 million years, when Papilionoideae diverged from Mimosoideae and Caesalpinioideae around 60 million years ago (Mya) [3]. Many legume species are important food crops, including peas, groundnuts, and beans.

Due to their economic importance, most legume genomic resource development has focused on the Papilionoideae subfamily. These include six published genome sequences: *Medicago truncatula* Gaertn. [4], *Lotus japonicus* L. [5], *Glycine max* (L.) Merr. [6], *Cajanus cajan* (L.) Millsp. [7], *Cicer arietinum* L. [8,9], and *Vigna radiata* (L.) R. Wilczek [10]. Comparative genomic analysis showed conserved syntenic blocks among Papilionoideae genomes, and loss of synteny with increased phylogenetic distance [11]. Previous analysis showed that two whole genome duplications occurred in the Papilionoideae lineage [6,12,13], and these played an important role in the rapid diversification of the species and the burst of new adaptive traits, such as nodulation [11,14]. This burst of new legume lineages and adaptations coincided with greater climatic aridity worldwide, marked by an increase in CO_2_, temperature, and humidity in the Pliocene [2,15].

In contrast to the well-studied Papilionoideae subfamily, little is known about the genome organization and evolution of Mimosoideae and Caesalpinioideae, which includes important economic and ecological members, such as in the clade mimosoids *Acacia*, *Prosopis,* and *Mimosa*. One genus in the Caesalpinioideae, Clade: Mimosoid, is *Prosopis* (called mesquites in N. America), which is particularly well adapted to severe desert environments, including via some of the world’s deepest and broadest root systems. *Prosopis cineraria* (L.) Druce (Arabic name Ghaf) thrives in harsh desert environments, including as the most abundant wild legume tree in the Arabian Peninsula, and is the national tree of the United Arab Emirates (UAE) (Quadri and Iyer, 2021). *P. cineraria* is also abundant throughout the middle east and arid regions of the Indian subcontinent [16]. *P. cineraria* leaves, pods, trunk, and bark are used by humans for various purposes. The unripened and ripened pods of the plant are eaten as vegetables or fruit, respectively [17]. Moreover, the leaf of the plant has been used as cattle feed in arid and semi-arid regions [18]. The trunk and branches of the tree are used as wood as well as fuel in desert regions [19]. This leguminous tree improves soil fertility by fixing atmospheric nitrogen and increasing available calcium and phosphorus [20,21].

*P. cineraria* is a long-lived phreatophyte, which means it produces a long tap root that will reach more than 30 m in search for underground aquifers [16]. *P. cineraria* is drought and salt tolerant, and is able to survive heat extremes that span both high (>45 °C) and low (<10 °C) temperatures that occur seasonally in desert regions. Unusually, it has a flowering mechanism in which blossoms appear during the hot dry season, where water availability is relatively scarce [16,18,22]. Genotypic variation has been investigated for the related mesquite species *Prosopis glandulosa* Torr., in which early flowering and fruiting were more abundant under water deficit and heat stress compared to well-watered plants [23].

In this study, we sequenced the genome of *P. cineraria* to a near-chromosomal level assembly. We annotated and characterized repeats and genes, with a particular emphasis on stress resistance genes, in the genome of *P. cineraria* in a comparative approach to other legumes. In the *P. cineraria* genome, we identified a dramatic burst of new gene creation by retrotransposition, and studied the expression of these retrogenes. We also increased our understanding of the genes and pathways involved in salt stress response using a transcriptomic approach. The genome resource and analysis of *P. cineraria* developed in this study provide insights into the biology and evolution of ecologically important desert trees, and generate an important new foundation for advancing the genetic improvement of legume crops. 

## 2. Results

### 2.1. Reference Genome Sequencing and Assembly

**Figure 1:**
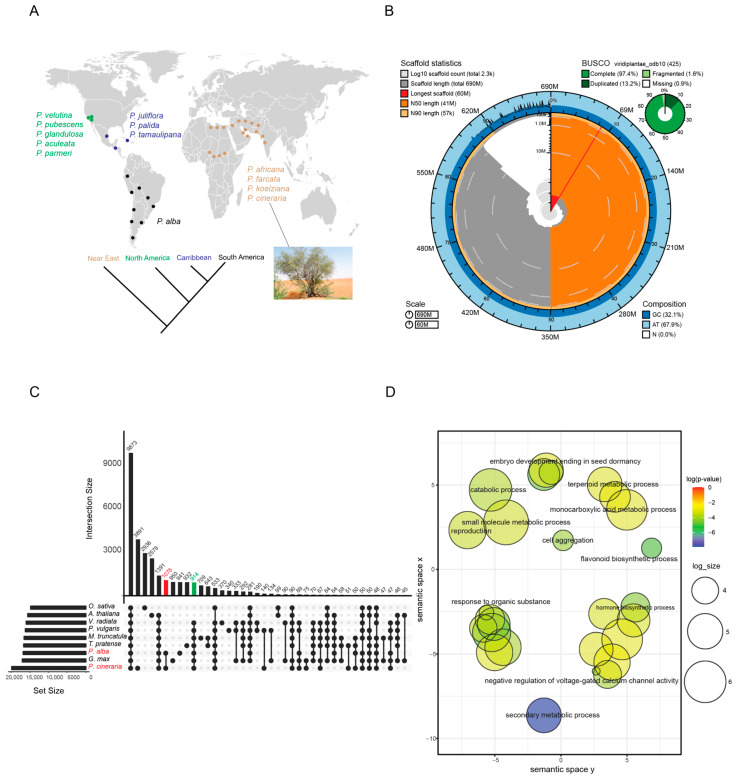
Distribution, genome assembly and orthology analysis of *P. cineraria*. (**A**) Geographic distribution of *Prosopis* species from around the world. *P. cineraria* is native to the Near East and Indian subcontinent, while other species are native to North and South America, and the Caribbean. (**B**) Blob Toolkit Snail plot describing assembly statistics. From inside to outside, cumulative scaffold count on log scale is depicted as light-gray spirals, and the changes in order of magnitude with white scale lines. The dark-gray segments show distribution of scaffold lengths, and the longest scaffold depicted in red was used to scale the plot radius. N50 and N90 scaffold lengths are highlighted in orange and light-orange rings, respectively. Blue and light-blue rings represent the percentages of GC, AT, and N in the genome assembly. (**C**) Orthologous group analysis of *P. cineraria* and *P. alba* from the mimosoid clade compared with other legumes are represented using UpsetR plot. Green bars represent groups shared with other legumes, while red bars are orthologous gene groups shared only by *P. cineraria* and *P. alba*. (**D**) GO enrichment analysis of the shared red bar plot orthogroups plotted using REVIGO, displaying the biological process. Each sphere represents a GO term colored by *p*-value in −log_10_ scale. The semantic similarity of these GO terms is represented by the position and distance among them. The log size is the logarithm of the number of terms present in each sphere.

For whole genome sequencing and de novo assembly, we selected a wild *P. cineraria* tree located in a desert environment in the United Arab Emirates (Figure 1A). In total, 213X (~997.5 million reads) Illumina reads and 65X (~7.7 million reads) PacBio long reads were used for the initial genome assembly (contig level) and error correction (Table S1). The first phase of genome assembly resulted in 691.6 Mb of assembled *P. cineraria* genome, which is ~98% of the estimated genome size of ~707 Mb estimated from k-mer analysis with Illumina shotgun reads (Figure S1). After the genome assembly, we obtained 3940 contigs with an N50 size of 649,960 bp (Table S2). In a second phase, we improved our genome assembly to a near-chromosomal level (pseudo chromosome) using ~306 million Omni-C reads for scaffolding. After scaffolding, we obtained 2271 contigs with a final genome assembly size of 691,857,940 bp (GC%: 32.08%). The scaffolding process lifted the assembly N50 value from ~0.64 Mb to ~41.4 Mb (~64 fold). The final genome assembly completeness was assessed using BUSCO analyses, which resulted in a BUSCO score of 99% (including complete (97.4%), fragmented (1.6%), duplicated (13.2%) and missing (0.9%) genes) (Figure 1B, Table S2). Additionally, we observed that the longest 14 scaffolds represent ~86% (594,687,247 bp) of the assembled genome, with sizes ranging from ~31.2 Mb to ~59.7 Mb (near-chromosomal level) (Table S3) [24]. From the total assembly, we separated six chloroplast-related sequences and the remaining 2265 scaffolds (includes 14 psuedochromosomes) were used for genome annotation process (Table 1).

### 2.2. Gene prediction and Annotation

The Braker-based gene prediction approach resulted in 84,842 gene models from the *P. cineraria* assembly. The predicted gene models were again refined using the Maker pipeline. In total, 76,554 CDS, 361 rRNAs and 664 tRNAs were identified in the genome (Table 1). The predicted genes were homology-searched against NCBI-NR and Uniport databases. A total of 55,325 (~72%) and 53,866 (~70%) of the predicted protein-coding genes were mapped against these two databases, respectively (Tables S4 and S5). In total, 9125 proteins were mapped against KEGG metabolic pathway genes (Table S6). We also identified 150 proteins (44 types of enzymes; Figure S3), which are involved in plant MAPK signaling (Table S7), 291 proteins (42 types of enzymes) involved in plant hormone signal transduction (Table S8 and Figure S4), 195 proteins (38 types of enzymes; Figure S5) involved in plant-pathogen interaction and 182 protein (21 types of enzymes; Figure S6) involved in phenylpropanoid biosynthesis from the KEGG analysis (Tables S9 and S10). 

Pfam domains, Gene Ontology (GO) terms and Interpro signatures were identified through InterProScan search. In total, 9132 transcription factors belonging to 58 plant transcription factor families were annotated from the gene models (Table S11). MYB-related (866), ERF (830), bHLH (693), NAC (531), TCP (527) and ZF-HD (458) are the most abundant transcription families found in *P. cineraria*. 

### 2.3. Orthologous Group Analysis

Predicted proteins in the assembled genome of *P. cineraria* and *Prosopis alba* Griseb. (*P. alba* data retrieved from NCBI database; WGS id: SMJV00000000.1) belonging to the mimosoid clade were compared to five members of the Papilionoideae subfamily within the legumes (Figure 1C) using *Arabidopsis thaliana* (L.) Heynh. and *Oryza sativa* L. as outgroup species. The comparison of the nine species in this study led to the identification of 33,123 gene families in total (Table S12). We identified 914 gene families that are specific to legumes. Not surprisingly, these legume-specific gene families include those enriched in genes involved in nodulation and nitrogen fixation, but we also found gene families enriched for loci in defense responses, flavanol biosynthesis, and gravitropism. In addition, we identified 1076 gene families that are specific to *P. cineraria* and *P. alba*, and these were enriched for terpenoid metabolic process, small molecule metabolic process, secondary metabolic process, embryo development ending in seed dormancy and response to abiotic stress stimulus (Figure 1D).

### 2.4. Genome Evolution in P. cineraria

**Figure 2:**
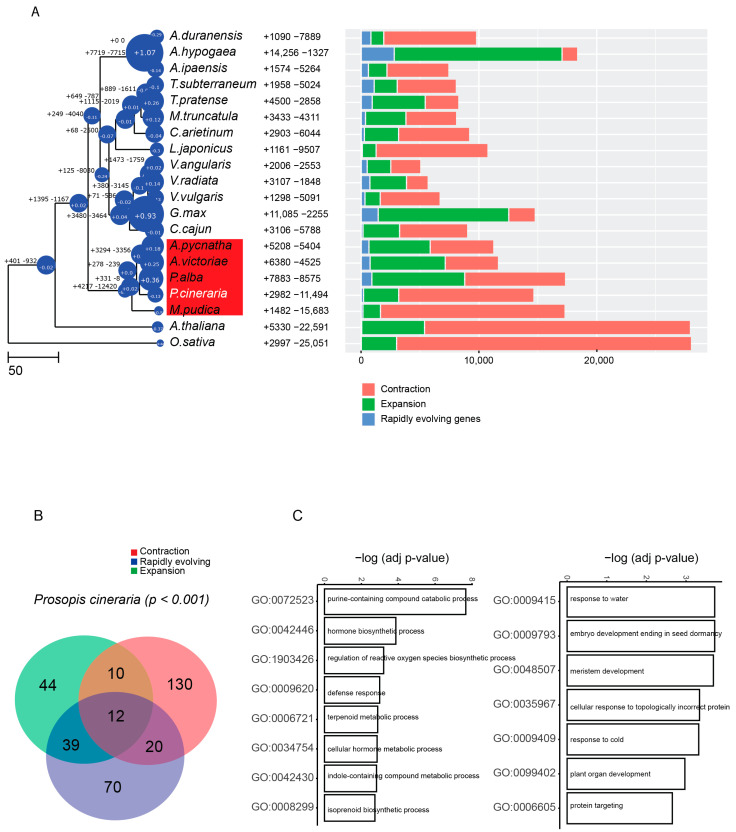
Genome evolution of *P. cineraria*. (**A**) Ultrametric tree of 18 legumes, including the mimosoid clade (highlighted by red box) and the two outgroups *A. thaliana* and *O. sativa*. CAFÉ analysis depicts total number of expanded and contracted gene families as well as rapidly evolving genes. The bubble on the node and leaf of the tree highlights the average expansion or contraction for each of the species, where a positive number depicts more expansion. (**B**) Venn diagram of top significant gene families (*p* < 10^−2^) that are expanded or contracted in *P. cineraria* and under positive selection. (**C**) GO term enrichments of expanded gene families that are under selection (**left**) and contracted gene families under selection (**right**).

We constructed a phylogenetic tree using single copy genes from the 18 species of legumes and outgroup species. We converted the phylogenetic tree to an ultrametric tree with divergence time estimates based on a calibration time of divergence between the two outgroups (Figure 2A). The tree shows an ~23 Mya divergence between *P. cineraria* and *P. alba* lineages, which is consistent with another molecular phylogenetic study by Cardoso and colleagues [2]. The large divergence time between these two *Prosopis* species may explain the weak synteny between the two genomes (Figure S7).

We observed in the *P. cineraria* lineage more gene family contractions than expansions (Figure 2A). *P. cineraria* gene families that show significant expansion (*p* < 10^−2^) are enriched for defense response loci such as disease resistance (NBS-LRR), for terpenoid and isoprenoid metabolism, as well as reverse transcriptase genes (Figure 2B,C). In contrast, we observed significant (*p* < 10^−2^) contraction of gene families enriched for PFAM genes related to plant organ developmental regulation and water response (Figure 2B,C). For instance, terpene synthase was found to be an orthogroup gene family that is significantly expanded in *P. cineraria* and *P. alba*, compared to other legumes, as well as compared to the outgroup species *A. thaliana* and *O. sativa*. On the other hand, water transport genes such as Major Intrinsic Protein (MIP), as well as flowering time and circadian rhythm coordination genes such as *AP2,* were contracted. The contraction of water response gene families is surprising, given the arid conditions where *P. cineraria* grows.

### 2.5. Comparative Analysis of Repeats, including Disease-Resistance Genes

**Figure 3:**
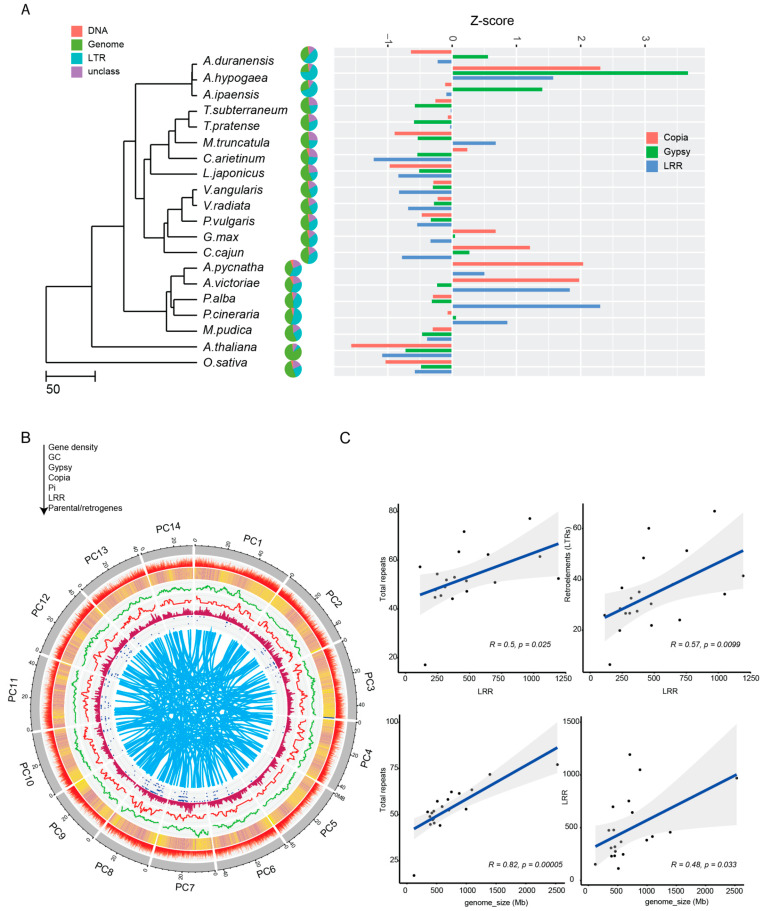
Comparative genome analysis of repeats, including disease resistance genes (NBS-LRR). (**A**) Pie chart of the percentage of DNA, LTR-retrotranspons, and unclassified repeats of the genome mapped onto the ultrameric tree. Z-score of the number of NBS-LRR, *copia*-like and *gypsy*-like LTR-retrotransposons across the phylogenetic tree. Positive values show increase in number while negative values depict a decrease. (**B**) Circos plot of co-localization of repeats and disease-resistance genes (NBS-LRRs) with different layers from outside to inside (black arrow direction) showing gene density followed by GC content, *gypsy*-like/*copia*-like repeats, (nucleotide diversity) in 20 Kb windows, and NBS-LRR distribution. Connected bands on the inside represents parental and retrogene distributions across the 14 longest scaffolds (pseudochromosomes) of *P. cineraria*. (**C**) Spearman correlation of disease-resistance genes (NBS-LRR) with total repeats as well as LTR-retrotransposons (top). Spearman correlation of genome size and total repeats as well as disease-resistance genes.

Our analysis indicates a significant expansion of disease-resistance gene families in *P. cineraria*; we also found specific gene families for pathogen resistance that were legume-specific. Our analysis found an intriguing connection between some of these disease-resistance genes and LTR-retrotransposon sequences. We observed this by mining the genome of *P. cineraria* for all types of repeats, and found that the genome has ~58.2% repeats, of which ~51.3% are LTR-retrotransposons (Figure 3A). As expected, LTR-retrotransposons are the most abundant repeat sequence in all species examined. Among the 18 species, *P. cineraria* and *P. alba* have the one of the highest fractions of LTR-retrotransposons in the genome (51.3 % and 41.4 %, respectively), followed by *G. max* (35.1%), *Trifolium pratense* L. (30.4%), *Phaseolus vulgaris* L. (26.6%) and the two outgroups *O. sativa* and *A. thaliana* with 26.7% and 8.5%, respectively. It should be noted that *Arachis* species have the highest LTR-retrotransposon contents fraction in these studied legumes (~60%). 

An abundant and important category of plant disease-resistance genes, the NBS-LRR genes, were mined in the same manner for each of these species in a comparative approach. We observed a great abundance of NBS-LRR gene candidates in *P. cineraria* and *P. alba* (753 and 1193, respectively), but only a few in others of these species (Figure 3A). There are significantly higher nucleotide diversity (π = 0.005) marks in these regions (Figure 3B), compared to the rest of the genome (π = 0.0018) (Wilcoxon test, *p* = 0.0003), a standard observation for this category of gene that has LRR regions under diversifying selection. Interestingly, we observed a frequent co-localization of disease-resistance genes (NBS-LRR) and LTR-retrotransposons. To test for co-localization, we calculated the average distance between LTR-retrotransposons and disease-resistance genes (NBS-LRR), and compared this to the overlap distance of LTR-retrotransposons to all genes in the genome. We observe that LTR-retrotransposons are in closer proximity to NBS-LRR genes compared to the rest of the genome (Figure S8, Table S13). We ran a permutation test (n = 1000, *p* < 0.05) using the R package regionR, which confirms that the co-localization of LTR-retrotransposons with NBS-LRR genes is not due to chance.

Finally, we observed a significant correlation between genome size and total repeat numbers (Spearman, R = 0.82, *p* = 10^−5^), as well as genome size and predicted NBS-LRR gene numbers (Spearman, R = 0.48, *p* = 0.033). In addition, NBS-LRR gene numbers are also significantly correlated with total repeat sequence numbers (Spearman, R = 0.5, *p* = 0.025) and LTR-retrotransposons sequences (Spearman, R = 0.57, *p* = 0.0099) (Figure 3C).

### 2.6. Retrogene Identification, Selection, and Activity in P. cineraria

A total of 55,325 annotated *P. cineraria* predicted genes encoding protein sequences were mapped against the near-chromosomal level genome assembly using RetroScan. We identified 785 candidate retrocopies that originated from 410 parental genes (Table S14). These retrocopies have an average length of 652.5 bp, mean pairwise identity of 0.64, and a coverage of 0.675. The average intron loss number compared to the parental gene is 2.9. The distribution of retrocopy numbers produced by the parental genes indicate that the majority (~73%) of parental genes only generated one copy, while a few generated more than five retrocopies (Figure S9). Interestingly, we observed only a small fraction (2.8%), which retains the parental gene open reading frame (ORF) and are classified as intact, whereas the majority are chimeric genes (57.3%) that possess nearby sequences, perhaps of a regulatory nature.

**Figure 4:**
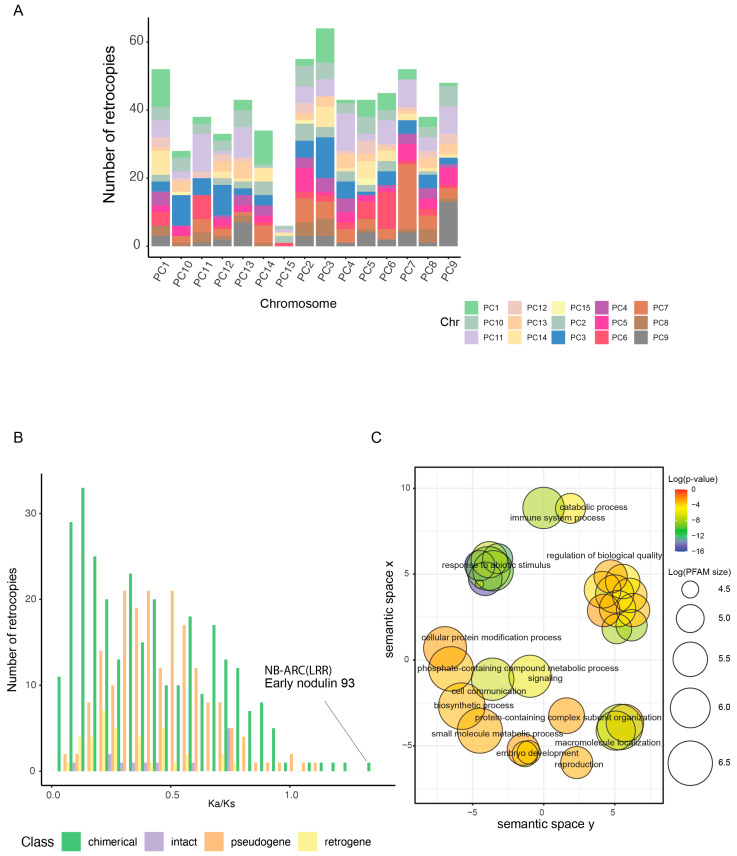
Retrogene identification and selection. (**A**) Genomic distribution of parental and retrogenes across 15 *P. cineraria* scaffolds (14 pseudochromosomes and one other scaffold). (**B**) The ratio of nonsynonymous substitutions to synonymous substitutions (Ka/Ks) of the parental to retrocopied genes for different classes of retrogenes. For Ka/Ks > 1, the Early nodulin 93 and NB-ARC genes are highlighted. (**C**) GO enrichment analysis of retrogenes displaying biological processes. Each sphere represents a GO term whose degree of enrichment is reflected in color on a −log_10_(P) scale. The semantic similarity of these GO terms is represented by the position and distance among them. The log size is the logarithm of the number of terms that are present in each sphere.

**Figure 5:**
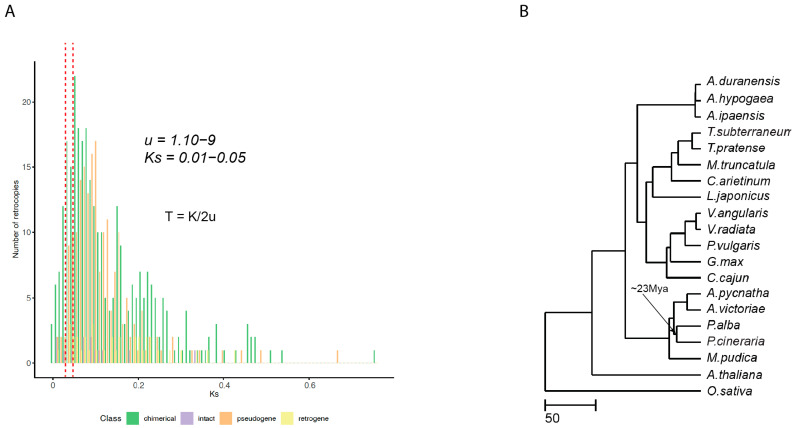
The timing of chimerical retrogene generation in *P. cineraria*. (**A**) Ks distribution of the different classes of retrogenes. The red dotted lines around Ks = 0.01–0.05 highlights the initial amplification. (**B**) Ultrametric tree highlighting the time of divergence of *P. cineraria* and *P. alba*, which overlaps with major amplification of chimerical retrogenes, using a mutation rate of 10^−9^ and using the formula (T = k/2u).

In our analysis, we detected 223 retrocopies that did not overlap with annotated genes. Nevertheless, 20 were intact and retained the ORF of their parental genes (Table S9). Finally, pseudoretrogenes, defined as those containing frame-shift mutations and/or premature termination codons, represent 27.4% of these retrocopies (Figure S10). Of course, nucleotide changes that do not frame-shift or prematurely terminate a peptide can also yield pseudogenes, so this 27.4% is a minimal estimate. All of these retrocopies were found to be normally distributed across the top 14 longest scaffolds (pseudo-chromosomes), with no bias toward any specific one (Figure 4A). Our results indicated a burst of chimerical retrogenes, which is illustrated with in the Ks distribution, reaching its peak between 0.02–0.05, which coincides with the divergence of the *P. cineraria* and *P. alba* lineages (Figure 5A,B).

To study the functionality of retrogenes, our first approach was to examine the ratio of non-synonymous (K_a_) to synonymous (K_s_) substitutions, comparing the K_a_/K_s_ ratio between retrogenes and their parental genes. We observed that the distribution of this ratio for chimerical retrogenes shows a peak at Ka/Ks < 0.2, and intact retrogenes have lower Ka/Ks than pseudoretrogenes (Figure 4B). Interestingly, we identified four chimerical retrogenes with Ka/Ks > 1 and these encode Early nodulin 93 (ENOD93 protein), a protein with an RNase H-like domain found in reverse transcriptase, an RPS5-like disease-resistance protein (NB-ARC domain), and a phosphoribulokinase/uridine kinase family gene. GO enrichment analysis for all retrogenes indicated that they include overrepresentation of genes involved in immunity, response to abiotic stimulus, small molecule metabolic processes, cell communication and biosynthetic processes (Figure 4C).

Using a second approach, we used our RNAseq transcriptome expression data for salt stressed tissues (see below) to validate the activity of retrogenes. We observed 401 expressed retrocopies (FPKM > 0) and if we consider the salt treatment versus control (FPKM > 0), the number is 355. However, the number of robustly expressed retrocopies (FPKM > 1) in control and salt environments was found to be 77 (Table S15). More chimerical retrogenes were expressed (40%), followed by intact retrogenes (8%), with pseudoretrogenes being the lowest fraction (2%).

### 2.7. Differential Gene Expression under Salt Stress

In total, ~673 million paired-end sequencing reads were generated from both root (control and treatment) and shoot (control and treatment) tissues (Supplementary Information D and E). After adapter and low quality trimming, we retained 94.3% of the reads. On average, around 85% of root reads and 96% of shoot reads mapped to the *P. cineraria* genome sequence (Supplementary Information F). For each sample, separate read count tables were generated from the alignments for differential gene expression (DGE) analysis. The relation between the samples (control and salt treatment) sequenced is estimated using principal component analysis (PCA) (Figure S11). We carried out transcriptome-based DGE analysis in shoot and root samples separately.

**Figure 6:**
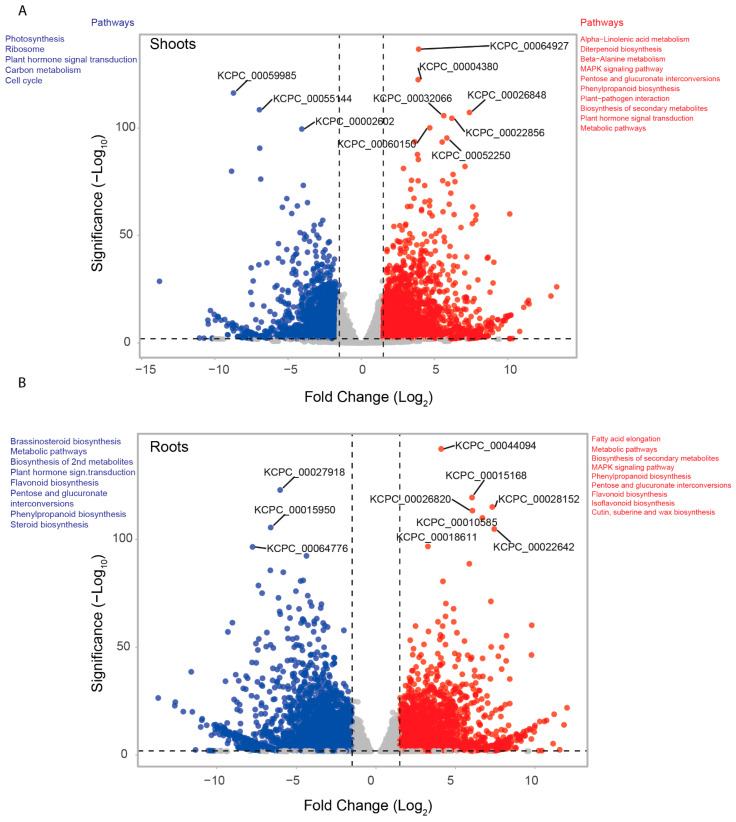
Differential gene expression in shoots and roots of *P. cineraria* under 250 Mm salt stress. (**A**) Volcano plot of shoots of up-regulated (red) and down-regulated (blue) at log_2_ fold change in 2 and −log_10_ significance. Gray dots represent neutral (relatively unchanged) genes. The names of the top up-regulated and down-regulated genes are indicated. Pathway enrichment analysis for the up-regulated (red) and down-regulated (blue) genes are depicted outside the volcano plot. (**B**) Volcano plot of roots of up-regulated (red) and down-regulated (blue) at log_2_ fold change in 2 and −log_10_ significance. Gray dots represent neutral (relatively unchanged) genes. Names of top up-regulated and down-regulated genes are indicated. Pathway enrichment analysis for the up-regulated (red) and down-regulated (blue) genes are depicted outside the volcano plot.

In shoot samples, we found 2065 up-regulated and 2108 down-regulated genes that differentiated control and salt stressed tissues (FDR < 0.05, fold change > +/−2) (Figure 6A). Important enzymes involved in plant hormone signal transduction (14 genes), MAPK signaling pathway (12 genes), plant-pathogen interaction (12 genes), starch and sucrose metabolism (9 genes), biosynthesis of amino acids (9 genes), and cysteine and methionine metabolism (8 genes) pathways were up-regulated in salt stressed shoot samples. In contrast, genes involved in photosynthesis (16 genes), ribosome (11 genes), plant hormone signal transduction (11 genes), carbon metabolism (10 genes) and cell cycle (10 genes) pathways were down-regulated in salt stressed shoot samples (Figure 6A).

In root samples, the DGE analysis revealed 2024 up-regulated genes and 2701 down-regulated genes in control vs salt stressed (Figure 6B). Genes involved in MAPK signaling pathways such as calmodulin, protein phosphatase 2C ERF1; ethylene-responsive transcription factor 1, basic endochitinase B, MAPKKK17/18, MAP kinase substrate 1 and 1-aminocyclopropane-1-carboxylate synthase 1/2/6 were up-regulated in salt stressed root samples. Moreover, genes involved in the phenylpropanoid biosynthesis pathway, plant signal transduction and sucrose-starch metabolism were up-regulated, while genes involved in the glycolysis pathway (10 genes), amino acid biosynthesis (10 genes), plant–pathogen interaction (8 genes), and plant hormone signal transduction (17 genes) pathway genes were down-regulated in root salt stressed sample.

## 3. Discussion

In this study, we generated a high-quality genome assembly of *Prosopis cineraria* by combining different NGS sequencing technologies (Illumina short read, PacBio long read, and Omni-C). We present a near-chromosomal level assembly with 14 pseudochromosomes and a high N50 of ~41 Mb compared to the reported genome assembly of a related South American species, *P. alba,* with N50 of ~248 Kb (NCBI genome data source).

Our orthologous gene group analysis highlights enrichment of genes involved in terpenoid metabolism that are specific to *P. cineraria* and *P. alba* in the mimosoid clade compared to other Papilionoideae legumes. The expanded terpene synthase gene family in this clade is consistent with the fact that terpenes are important bioactive substances in *Prosopis* spp. [25]. As a gatekeeper to plant terpenoid chemical diversity and evolution, these genes are driven by selection to adapt to biotic and abiotic stresses [26,27,28]. In addition, their expansion often results in lineage-specific pathways or products [29,30,31]. The fact that *P. cineraria* terpenoid (isoprenoid) bioactivity is diverse may partly explain why human exploitation of this species is widespread in the Near East and Indian subcontinent [16,32].

The innate immune system in plants uses a repertoire of receptors that sense pathogens and trigger an immune response. A big fraction of these receptors is from the NBS-LRR gene family. *P. cineraria* and *P. alba* have amplified NBS-LRR gene numbers compared to other legumes, and this is significantly correlated with both the abundance and location of LTR-containing retroelements. Our evidence for retro-amplification of NBS-LRR genes is consistent to what is known in some other plants [33,34,35]. In addition, the high diversity of NBS-LRR disease-resistance genes in *P. cineraria* suggests balancing selection acting on these genes. One possible explanation is that the woody nature and long lifespan of *P. cineraria* may lead to greater exposure to pathogens and less meiotic recombination events to generate novelty. Thus, more NBS-LRR genes could provide a broader array of pathogen sensors as well as promote frequent unequal recombination to amplify and diverge these genes [36,37,38].

It has been surmised that by recruiting new proteins, chimerical retrogenes are likely to drive genetic innovation and adaptive evolution [39]. Our analysis identifies a burst of chimerical retrogenes in the *P. cineraria* genome. In general, nuclear sequences are assumed to have a mutation rate on the order of 10^−8^ to 10^−9^ substitutions/site/generation [40]. Since we do not have a genome-wide mutation rate estimate for *P. cineraria*, we use an estimate of ~10^−9^ from woody long-lived trees such as conifers [41]. The Ks distribution of chimerical retrogenes in *P. cineraria* peaks between 0.02–0.05, which is equivalent to ~10–25 Mya divergence, suggesting that a large fraction of these retrogenes originated in this evolutionary time period.

Interestingly, this burst of chimerical retrogene generation coincides with our estimate of the divergence of *P. cineraria* from shared lineage with *P. alba,* as well as a similar estimate by Cardoso and coworkers [2]. Our results also indicate that the majority of chimerical retrogenes are under purifying selection (Ka/Ks < 1), which means that they are under functional constraint compared to pseudogenes (Figure 3C). Enrichment analysis of these chimerical retrogenes highlighted top GO genes involved in immune system processes, responses to abiotic stimulus, and biosynthetic processes (Figure 4C). Interestingly, we also detected a few chimerical retrogenes with Ka/Ks > 1 (Figure 3C), suggesting that they have been under positive selection and, thus, may have evolved into new functions. One of these genes encodes an NB-ARC domain containing protein (disease-resistance gene). A genome-wide study in peppers concluded that retroduplication played a major role in the expansion of disease-resistance genes in the species [42]. In addition, previous studies suggested a correlation between transposable elements mediated gene duplication and specific disease-resistance gene family expansion in plants [43,44]. For instance, NLR (nucleotide binding and leucine-rich-repeat proteins) are among the highly amplified gene family, which provide functional disease-resistance loci in plants [33,34,35]. Comparative genomic analysis suggests also a possible co-evolution between long terminal repeat and retrotransposons (LTR-retrotransposons) and NLR, and this is because of often genomic co-localization [34,45,46]. A second gene apparently under positive (“diversifying”) selection is a homologue of early nodulin 93, which could be crucial for nitrogen use efficiency in legumes. Alignment and homology predictions show structural similarity between Arabidopsis Early nodulin protein with other subclasses that is expressed very early in developing nitrogen-fixing root nodules of legumes *(Pisum sativum* L., *Vicia sativa* L., *M. truncatula*, and *G. max*) [47]. Moreover, the expression of this gene in a non-legume, rice, was shown to improve yield under limited nitrogen conditions [48]. Moreover, we show that many of these chimerical retrogenes are transcribed. These results in *P. cineraria* suggest that retrotransposition contributed to the origin of new genes that have played a role in evolutionary adaptation.

The transcriptome analysis performed under control and salt stress conditions in both root and leaf samples revealed key genes that are possibly related to salt stress response. In leaf samples, a pentatricopeptide repeat-containing (PPR; KCPC_00000076) protein gene was up-regulated almost 13-fold (log_2_; FDR value: 8.49 × 10^−25^) in salt stressed samples. In rice, PPR gene function was validated to enhance both drought and salt stress tolerance [49]. An endoglucanase gene (KCPC_00045262) was up-regulated in both leaf (~13 fold; *p*-value: 1.14 × 10^−20^) and root (~7.2 fold; 0.03 *p*-value) samples during the salt stress. In maize, this gene was reported to be correlated with cell-wall extensibility under salt stress [50]. Another gene, polygalacturonase (KCPC_00051679), is mainly involved in cell wall stability and was up-regulated in stress conditions in both leaf and root tissues. Overexpression of this gene decreases the cell wall pectin content and helps plant survival during various stress conditions [51]. Finally, another up-regulated gene is a wall-associated receptor kinase (WAK) (KCPC_00068206), which mainly acts as a cell wall sensor in plant stress response pathways. It controls MPK3 and MPK6 pathways and provide stress tolerance to plants [52]. Along with these at least partly expected DEG results, future studies of the DEGs of unknown or barely known function will be particularly interesting and valuable.

Pathway enrichment analysis in shoot and root highlight tissue specific expression of genes related to terpenoid (isoprenoid) and plant-pathogen defense in shoots, but not found in roots. This suggest that biosynthesis of these compounds is restricted to the shoots, which could be tightly linked to internal and external stimuli regulation to fine-tune terpenoid development to mediate proper interaction with the environment [27,53,54].

## 4. Materials and Methods

### 4.1. P. cineraria Sample Collection and Sequencing

For the reference genome assembly, fresh leaves of *P. cineraria* were collected from one tree (~15–20 years old) growing in the desert of Sweihan area (Figure 1A; 24°17′18.3″ N 55°43′36.2″ E), Al Ain, Abu Dhabi Emirates, UAE. Genomic DNA was extracted from the green leaves using a modified Cetyl trimethylammonium bromide (CTAB) method (detailed DNA isolation method is described in Supplementary Information A). The quantity of the DNA was determined by NanoDrop 2000 (Thermo Scientific, Waltham, MA, USA), and the quality was confirmed in a 1% (*w*/*v*) agarose gel.

To assist gene annotation, RNA was extracted from leaves, roots, or flowers using a modification of a previously published method [55] (the detailed RNA isolation method is described in Supplementary Information B). The isolated RNA concentration was estimated by NanoDrop 2000 (Thermo Scientific, Waltham, MA, USA), and quality was checked by 1.2% agarose gel. The extracted RNA was sent to the Yale Center for Genomic Analysis (YCGA) for library preparation and sequencing on Illumina Hiseq 2000 (Illumina, San Diego, CA, USA).

### 4.2. Reference Genome Assembly and Genome Size Estimation

We generated shot-gun sequencing reads (Illumina-based) and long sequencing reads (PacBio-based) data from the isolated *P. cineraria* DNA. Illumina compatible libraries (insert size 300–600 bp) were generated and sequenced (150 bp PE chemistry) on the Illumina NextSeq 2000 platform following Illumina chemistry and instructions. The raw Illumina data were trimmed (low quality and adapter contamination) using Trimmomatic v.3 [56] and further quality of trimmed reads was confirmed using the FastQC tool [57]. Continuous long reads were produced from >20 kb insert SMTRbell libraries (SMRTbell Template Prep Kit 1.0) on PacBio RSII platforms.

The Illumina shotgun PE reads generated were used for genome size estimation using a k-mer-based method. We generated k-mer count (19-mers, 21-mers and 23-mers) as well as k-mer histogram files from trimmed and cleaned Illumina reads using Jellyfish v.2.3.0 [58]. Based on the k-mer histogram information, the theoretical genome size of *P. cineraria* was estimated using GenomeScope v.1 online tool [59].

Initial genome assembly was carried out by CANU (v.1.6) [60] software using Pacbio long reads with default parameters. Briefly, all raw long reads were corrected with CANU correction and then assembled with CANU assembly. Assembled contigs were polished using Arrow (PacBio) on Pacbio reads. Furthermore, polished contigs were error corrected using Illumina reads with Pilon v.1.23 [61] program.

For scaffolding the genome into a near-chromosomal level, we used Omini-C libraries (Dovetail) generated using the NEBNext Ultra enzymes kit and sequenced on the Illumina HiSeqX platform (target to obtain ~30X coverage). We used HiRise [62] for scaffolding the primary contigs. During the scaffolding process, Omini-C library PE reads were aligned to the primary contigs using bwa v.0.7.7 [63]. The aligned files were further processed using the HiRise program. A likelihood model for genomic distance between aligned read pairs (MQ > 50) was inferred, and this model was used to scaffold the genome as well as to repair the mis-joins found in the initial contig assembly. The assembled genome completeness was assessed using BUSCO v.4.1.4 (db: viridiplantae_odb10) tool [64].

### 4.3. Gene Prediction and Genome Annotation

We followed both a homology-based and a de novo approach for gene prediction. Initial gene prediction was performed using Braker v.2.1.5 pipeline [65]. We used 488,097 protein sequences from 11 different plant species (Supplementary Information C) and transcriptomes generated from root, shoot and flowers of *P. cineraria* for training gene models (Supplementary Information D). The transcriptome reads were aligned to the masked genome using HISAT2 v.2.1 program [66] and aligned BAM files were used for transcriptome-based gene prediction. Program gth v.1.7.1 [67] was used for plant protein alignment against assembled genome. Both transcriptome and protein alignment hint files were used for GeneMark v.4.61 gene prediction [68] and for Augustus v.3.3.3 species model creation and gene prediction [69]. We carried out a second round of gene prediction using the Maker v.3.01 pipeline [70]. With Maker, gene models were predicted using SNAP [71], GeneMark, Augustus and EVM v.1.1.1 [72]. Detailed gene prediction workflow is illustrated in Figure S2. All rRNA and tRNA genes were predicted using RNAmmer v.1.2 [73] and tRNAscan-SE v.2.0.6 [74] programs, respectively.

The predicted protein-encoding genes were used in similarity searches against NCBI-NR [75] and UniProt protein [76] databases. The Pfam [77], InterPro [78], and Gene Ontology (GO) information [79] for the predicted genes was obtained with an InterProScan v.5.54 search [80]. BloobTools2 v.2.5 [81] was used to generate a snail plot of genome assembly statistics and predicted protein-encoding gene completeness from BUSCO. From the predicted genes, metabolic pathway enzymes were annotated using KEGG-KAAS [82,83]. Moreover, predicted proteins were similarity searched against a plant transcription factor database (PlantTFDB 2.0) [84] to identify possible transcription factor-encoding genes.

### 4.4. Orthogroup Analysis

Orthofinder2 v.2.3.12 [85] was used to identify orthologous groups in *P. cineraria*, *P. alba* (SMJV00000000) from the mimosoid clade in comparison with other legumes, such as *G. max* (GCA_000004515), *T. pratense* (GCA_020283565), *M. truncatula* (GCA_003473485), *V. radiata* (GCA_000741045), and *P. vulgaris* (GCA_000499845). We used *A. thaliana* and *O. sativa* as outgroups. All these genomes are available from the NCBI genome database. Orthogroup presence and absence were summarized in an UpsetR [86] plot. Orthogroups that were shared across all legumes, but not with *P. cineraria* and *P. alba*, were extracted and GO enrichment was summarized using REVIGO [87]. A similar analysis was performed with Orthogroups that are unique for *P. cineraria* and *P. alba* but not shared with other legumes.

### 4.5. Phylogenetic Analysis and Divergence Times

To estimate the evolutionary relationships among 18 legumes species (https://www.ncbi.nlm.nih.gov, accessed on 2 March 2022), including the outgroups *A. thaliana* and *O. sativa*, Orthofinder2 was used on all protein-coding genes. Single copy Orthogroups mined by Orthofinder2 were used to construct a phylogenetic tree. R8s [88] was used to convert the newick tree to an ultrametric tree, using the *A. thaliana* and *O. sativa* median divergence time from TimeTree (http://www.timetree.org, accessed on 10 March 2022), which is ~152 Mya, and the number of sites (1,423,440) that went into generating the species tree.

### 4.6. Genome Evolution of P. cineraria

All expansions, contractions, and rapidly evolving gene families from the 18 legumes with the two outgroups were determined using Orthofinder2. Species-specific gene family’s expansion and contractions were identified using CAFÉ v.5 [89] programs. We ran Café Fig (https://github.com/LKremer/CAFE_fig, accessed on 28 March 2022) to extract the top significant expansions, contractions and under selection at *p* < 10^−2^. A Venn diagram for the intersection of expanded, contracted. and under selection gene families was generated as well as GO term enrichment. Barplots for gene families expanded, contracted, and under selection were generated using R package GOplot [90].

### 4.7. Comparative Analysis of Repeats, including NBS-LRR Disease-Resistance Genes

To build a de novo repeat library for *P. cineraria*, we used ab initio predictions of three available programs, RepeatModeler v.2.0.1 [91], for all classes of repeats, plus LTRharvest and LTR retriever for the identification of LTR-retrotransposons [92,93]. LTR retriever was used to extract LTR-retrotransposon models from the structural annotation of LTRharvest. LTRharvest was used at a 90% identity of LTRs as a unique family threshold, with a requirement for the presence of the canonical terminal motifs, 5’-TG and 3’-CA. De novo repeat prediction from the *P. cineraria* genome was carried out using RepeatModeler tool. From the predicted repeat dataset, possible proteins and transcripts that were related to repeats were removed from the assembled genome prior to gene prediction using default parameters to run RepeatMasker v.4.1 tool [94]. Identical methods for repeat annotation were applied for other genomes used in the orthogroup and phylogenetic analysis.

NBS-LRR (Nucleotide Binding-Site, Leucine-Rich Repeat) disease-resistance genes were mined using NLgenomesweeper [95]. This tool uses the genome sequences instead of the annotated proteins to annotate the most conserved domain, NB-ARC (nucleotide binding adapter shared by APAF-1, R proteins, and CED-4), of NBS-LRR genes using a Blast suite. The NBS-LRR genome coordinates were generated as well as InterProscan ORF and domain for further manual curation. A Circos plot [96] was generated with gene density, GC content, abundant LTR repeats, NBS-LRR distribution, and nucleotide diversity across the genome.

### 4.8. Retrogene Identification and Expression in P. cineraria

To identify retrogenes in the genome of *P. cineraria*, we used the RetroScan [97] pipeline. This tool was used because of its accuracy and low rate of false positives. We used shinyapp (https://github.com/Vicky123wzy/RetroScan, accessed on 20 January 2022) for visualization of the results and for generation of retrocopy distribution, localization, and expression under salt stress as well as the selective force acting upon retrogenes in the genome. GO enrichment analysis for retrogenes was summarized using REVIGO. Filtered raw reads from the salt experiment in triplicates was used for retrogenes activity analysis.

### 4.9. Differential Gene Expression in P. cineraria

For the salt stress experiment, *P. cineraria* seed were collected from the same tree used for genome sequencing. Collected seed were sterilized with 30% (*v*/*v*) bleach (Clorox) for 15 min and washed three times with sterile Milli-Q water. They were placed on 1/2 strength MS agar (0.7% *w*/*v*) medium and allowed to grow at 25 °C in a growth chamber. Five-week-old seedlings were transferred to hydroponic trays and acclimatized for five weeks. Later, the seedlings were subjected to salt stress (48 h) by replacing the media in the trays with fresh medium with and without 250 mM salt stress for test and control, respectively. Three replicates of roots and shoots were collected from the control and treated samples and were snap-frozen for transcriptome expression analysis (Supplementary Information E). The samples were sent to Novogene for RNAseq sequencing (Illumina).

The quality of raw transcriptome reads was assayed using the FastQC program. Adapters and low quality regions found in the reads were trimmed using Trimmomatic tool. Here, we followed a reference-based transcriptome approach for the gene expression analysis [98]. The reference genome index was created, and the trimmed reads were aligned against the reference genome using the HISAT2 tool. Aligned SAM files were converted into sorted BAM files using Samtools v.1.10 [99]. From the sorted BAM files, the transcriptome assembly was carried out using Stringtie [100] and read counts corresponding to each gene were extracted for differential gene expression (DGE) analysis. We used DESeq2 [101] for the DEG analysis, and FDR value < 0.05 and fold change in at least +/−2 were considered to be significant for up-regulated and down-regulated genes.

From the up-regulated and down-regulated genes that were differentially expressed, GO enrichment was predicted using dcGO [102] and the enriched GO terms were visualized using the REVIGO tool. The pathway enrichment analysis of the DEGs were performed using ShinyGo v.0.75 [103].

## 5. Conclusions

*Prosopis cineraria* is among a small number of native trees that thrives in arid environments. It is used extensively in desert societies for its social, economic, ecological, and medicinal values. Given that the rate of desertification has been increasing in the past many decades, understanding desert plants’ survival strategies may help support translational agronomics, breeding, genetics, and genomics for crop improvement. The released *P. cineraria* genome will provide a key genomic resource and assist identification of the adaptive responses of genes that might be used in the enhancement of crop legumes.

## Figures and Tables

**Table 1:** *P. cineraria* genome assembly statistics.

Features	Values
Total scaffolds	2265
Total genome size	691,392,202 bp
Pseudochromosome	14
Pseudochromosome coverage	~86%
(A + T) percentage	67.8%
(G + C) percentage	32.1%
N percentage	2.44%
Min sequence length	4999 bp
Max sequence length	59,799,197 bp
Average sequence length	305,250.42 bp
N50 length	41,482,946 bp
L50 number	8
Repeat %	58%
Number of genes	76,554
Number of exons	344,680
Number of rRNA genes	361
Number of tRNA genes	664

## Data Availability

The sequencing data (Illumina shotgun genomic reads, PacBio long reads, Omini-c reads and transcriptome reads) generated during this study are deposited in NCBI-SRA database under the Bioproject id: PRJNA838117. The assembled genome and predicted protein were deposited in zenodo data repository, the data can be accessed via web link: https://doi.org/10.5281/zenodo.6720540.

## Supplementary Materials

The supporting information can be downloaded at: https://www.mdpi.com/article/10.3390/ijms23158503/s1.
