# Supplementary material for: The Genome of the Mimosoid Legume Prosopis cineraria, a Desert Tree
Source: Int J Mol Sci. 2022 Jul 31;23(15):8503. doi: 10.3390/ijms23158503 (PMC9369113; doi:10.3390/ijms23158503)
Supplement: Supplementary file 1 [file ijms-23-08503-s001.zip › PC_Supplementary_figures_final_26_07_22.pdf]

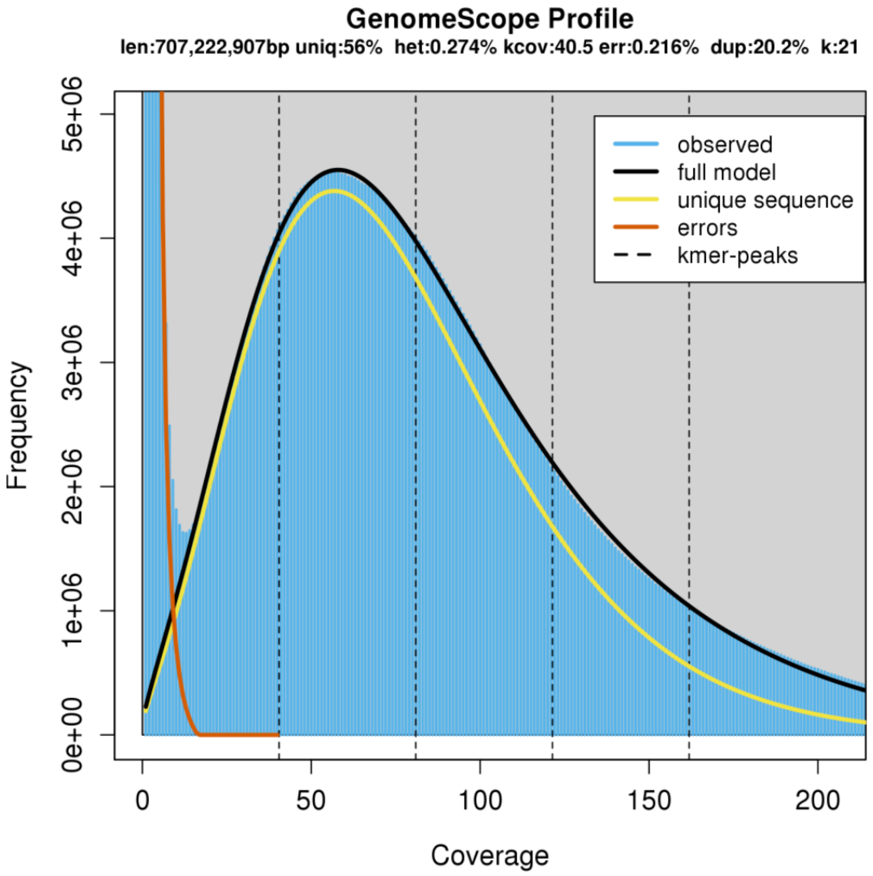

Results

GenomeScope version 1.0
k = 21

property	min	max
Heterozygosity	0.265354%	0.282183%
Genome Haploid Length	704,082,552 bp	707,222,907 bp
Genome Repeat Length	309,963,855 bp	311,346,359 bp
Genome Unique Length	394,118,697 bp	395,876,548 bp
Model Fit	94.9425%	98.5894%
Read Error Rate	0.216386%	0.216386%

Sup Figure S1. Genome size estimation based on 21-mers.

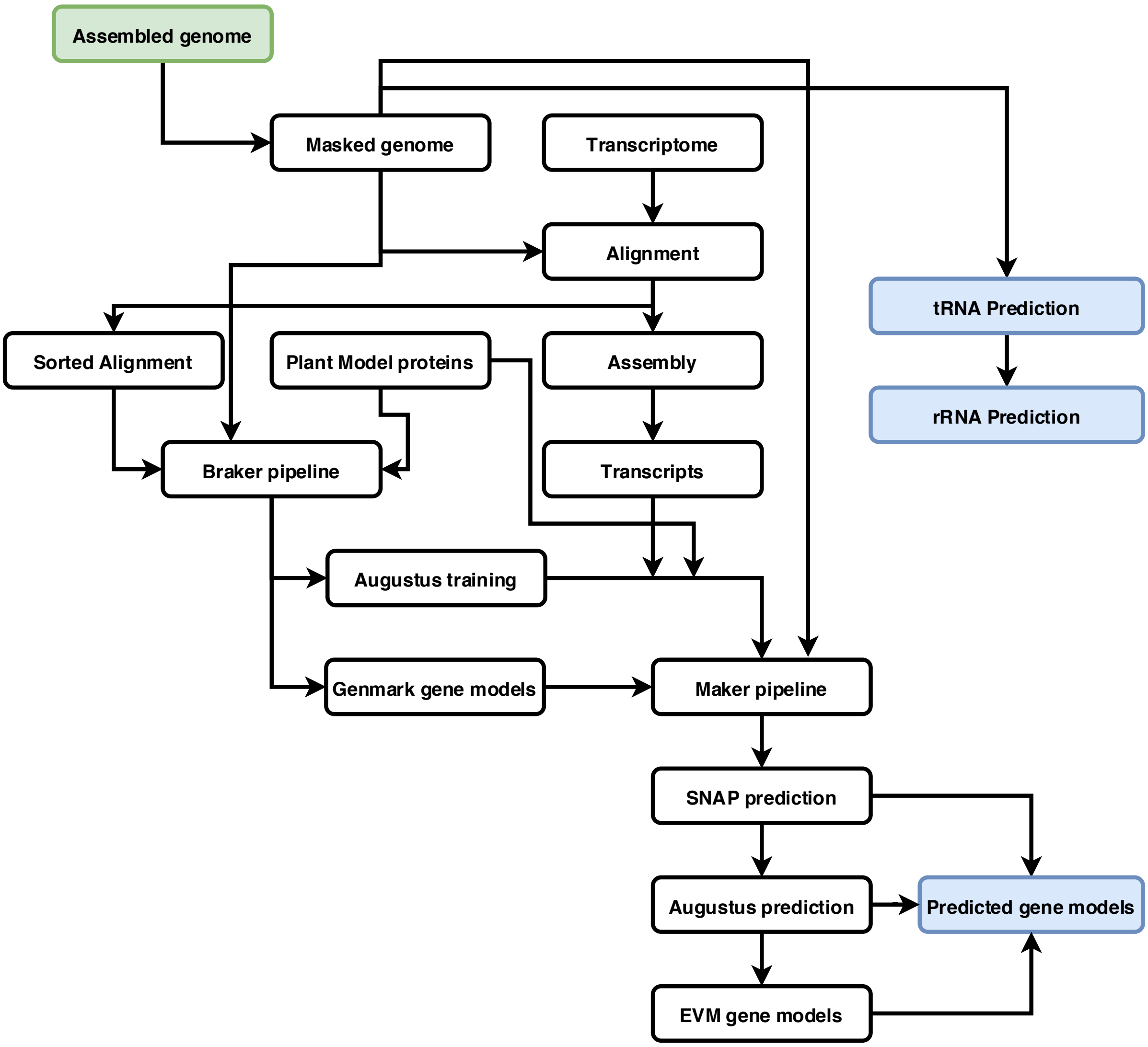

Sup Figure S2. Genome annotation workflow

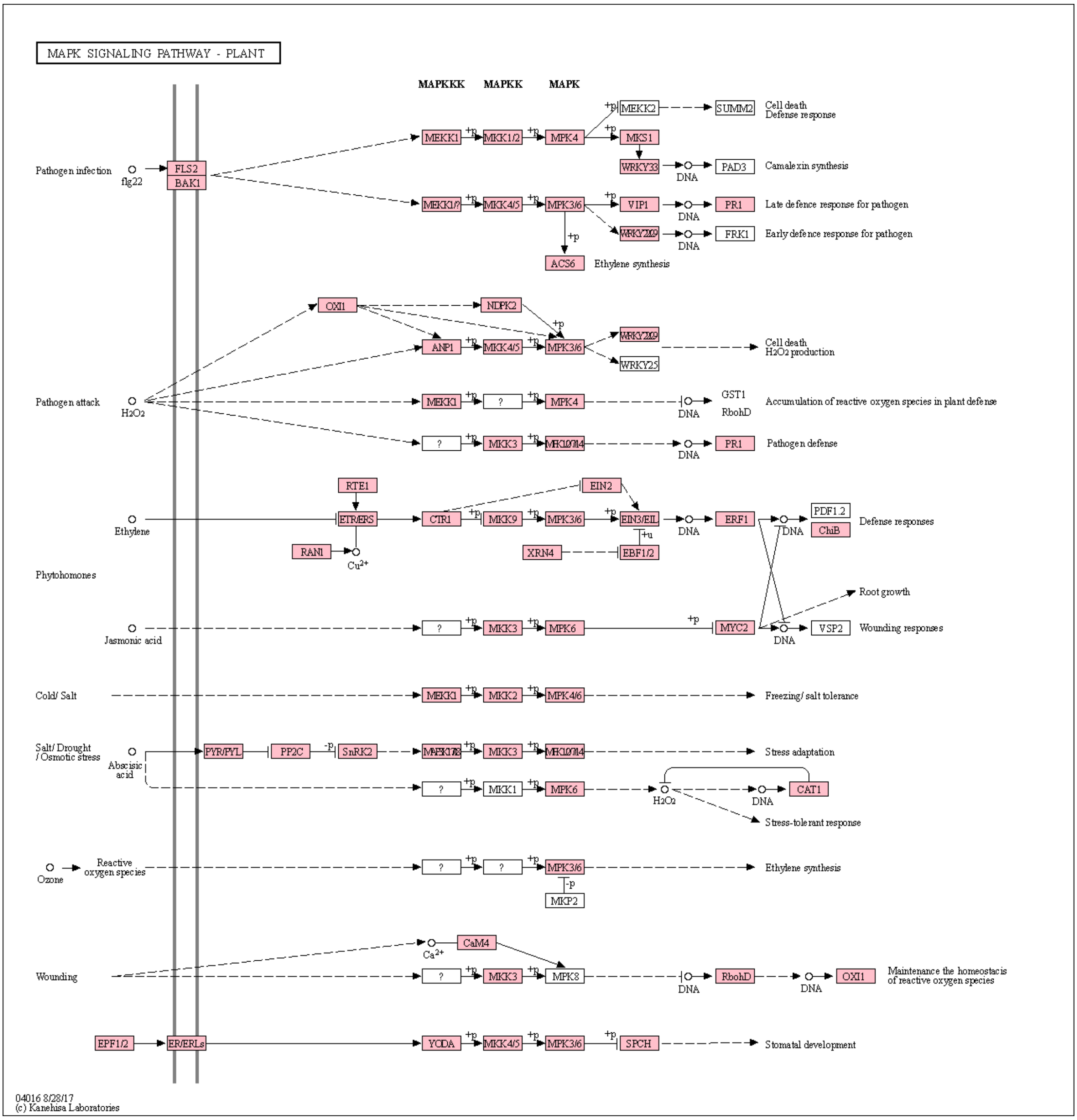

Sub Figure S3. MAPK plant pathway; identified enzymes are highlighted in the reference pathway.

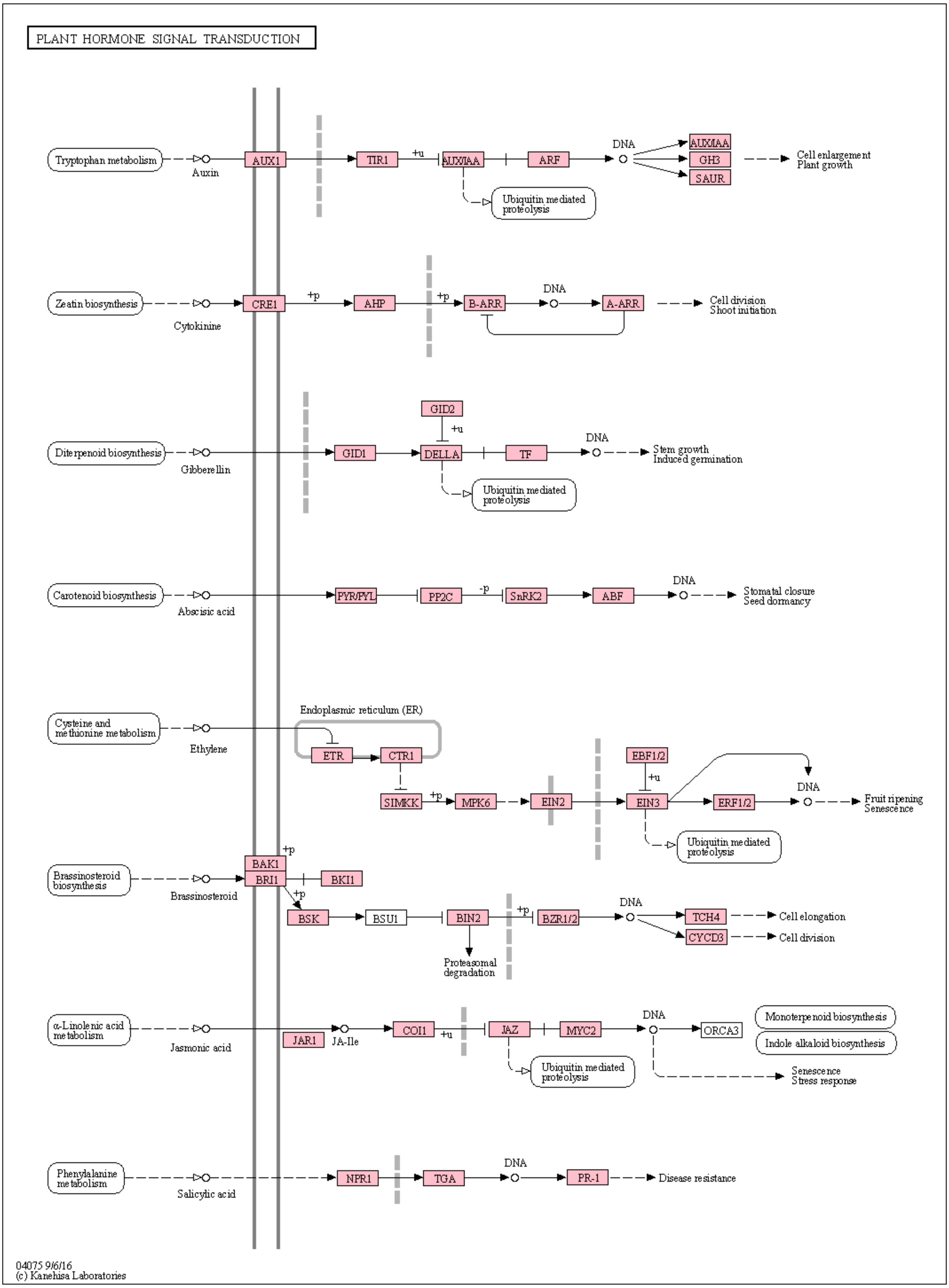

Sup Figure S4. Plant hormone signal transduction pathway; identified enzymes are highlighted in the reference pathway.

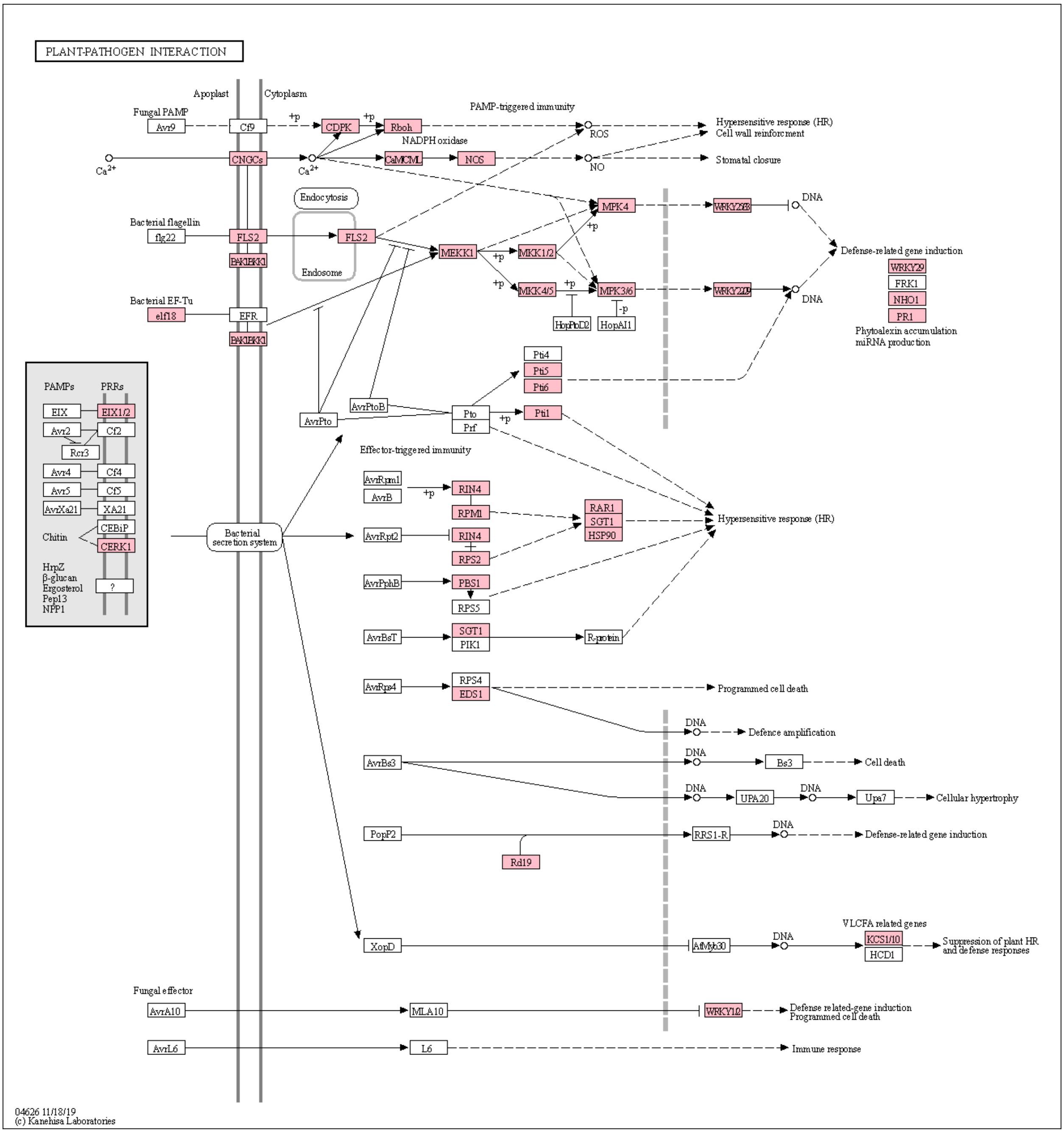

Sup Figure S5. Plant-pathogen interaction pathway, identified enzymes are highlighted in the reference pathway.

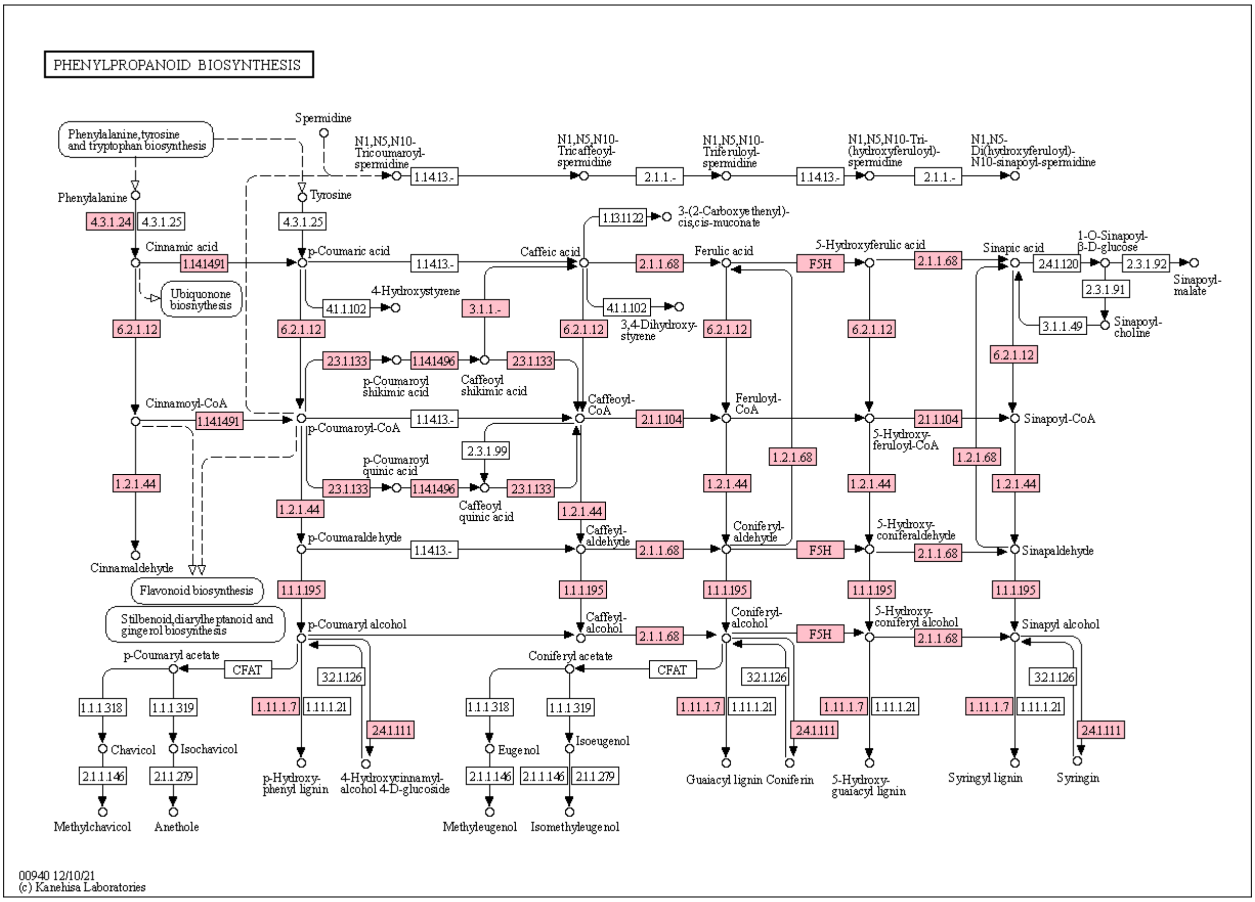

Sup Figure S6. Phenylpropanoid biosynthesis pathway, identified enzymes are highlighted in the reference pathway.

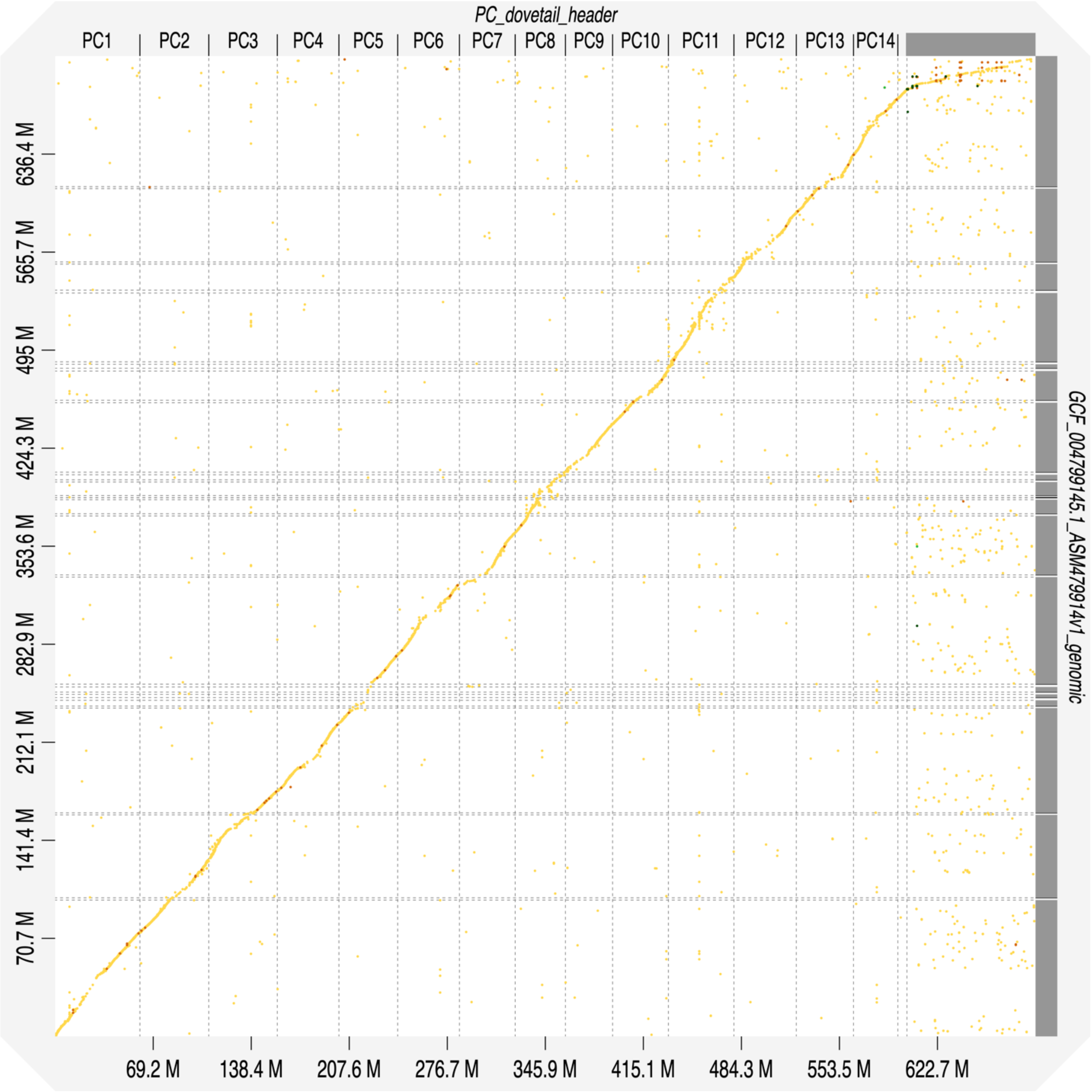

Sup Figure S7. whole genome syntheny plot of *P. cineraria* and *P. alba*.

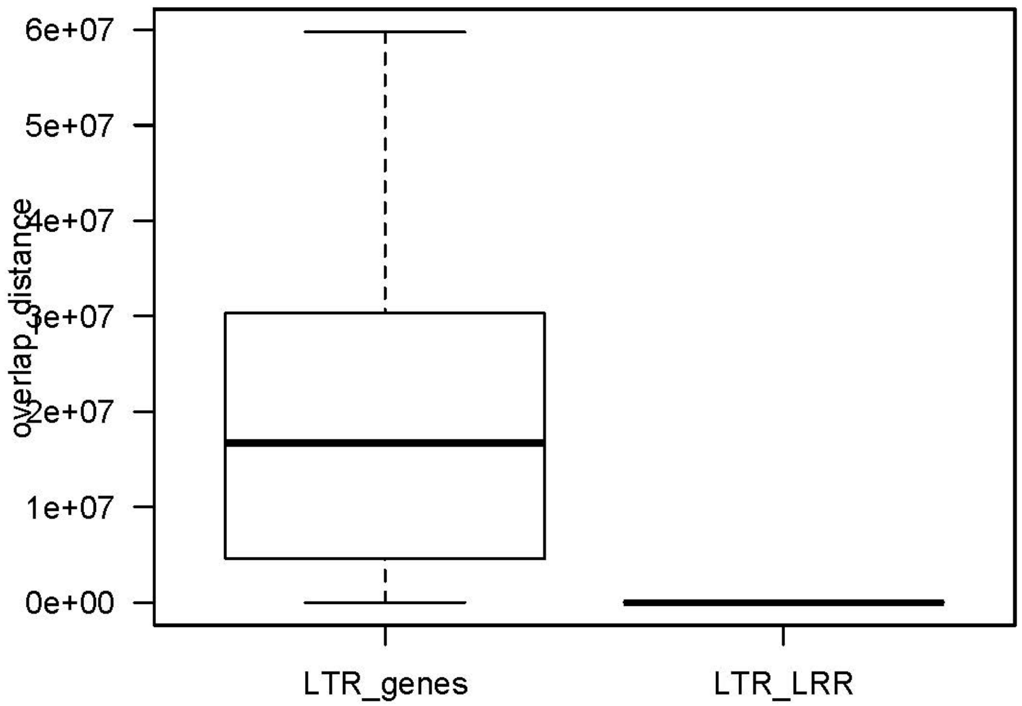

Sup Figure S8. Boxplot represents results of bedtools overlap distance between LTR and LRR in comparison to other genes

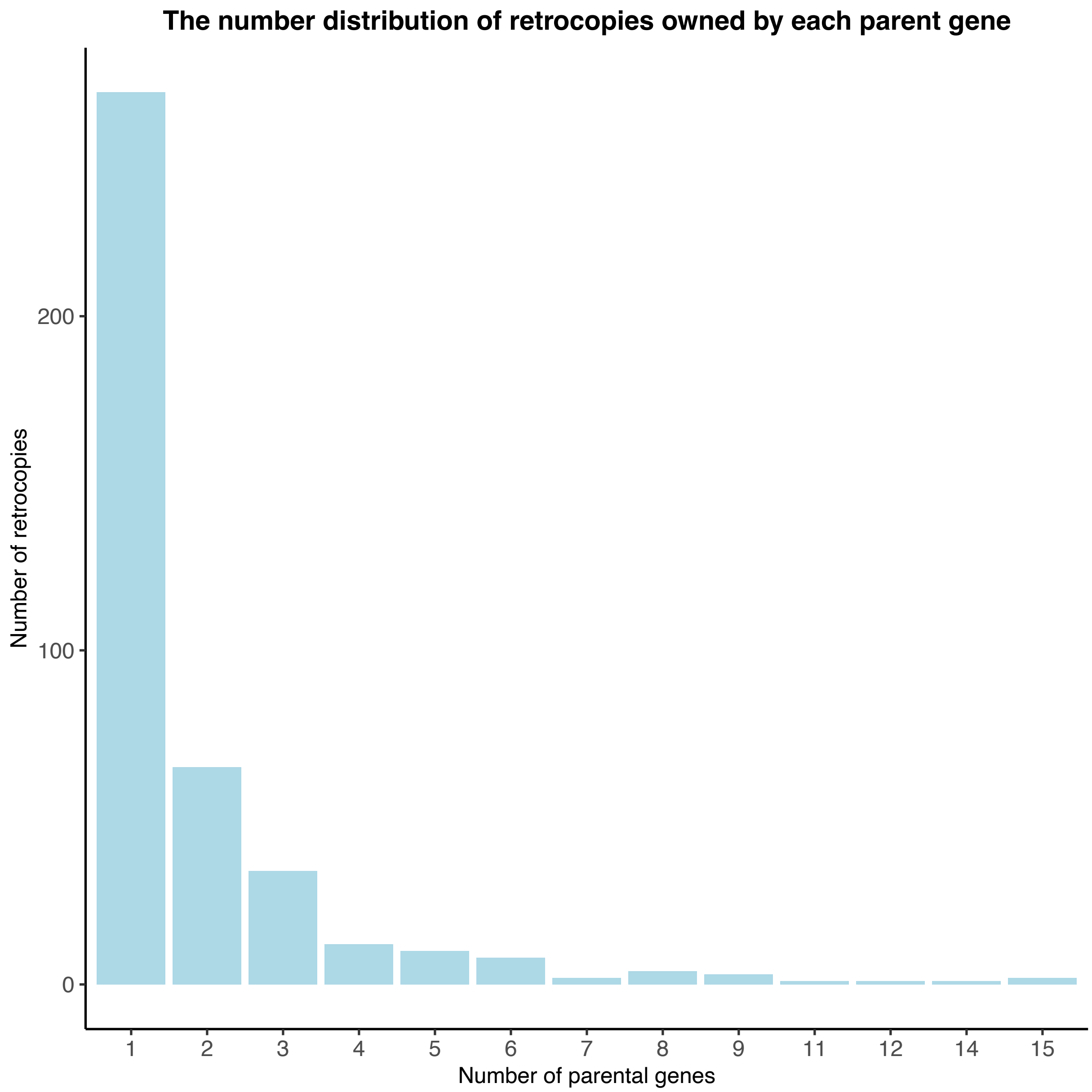

Sup Figure S9. *P. cineraria* retrocopy numbers per parental genes.

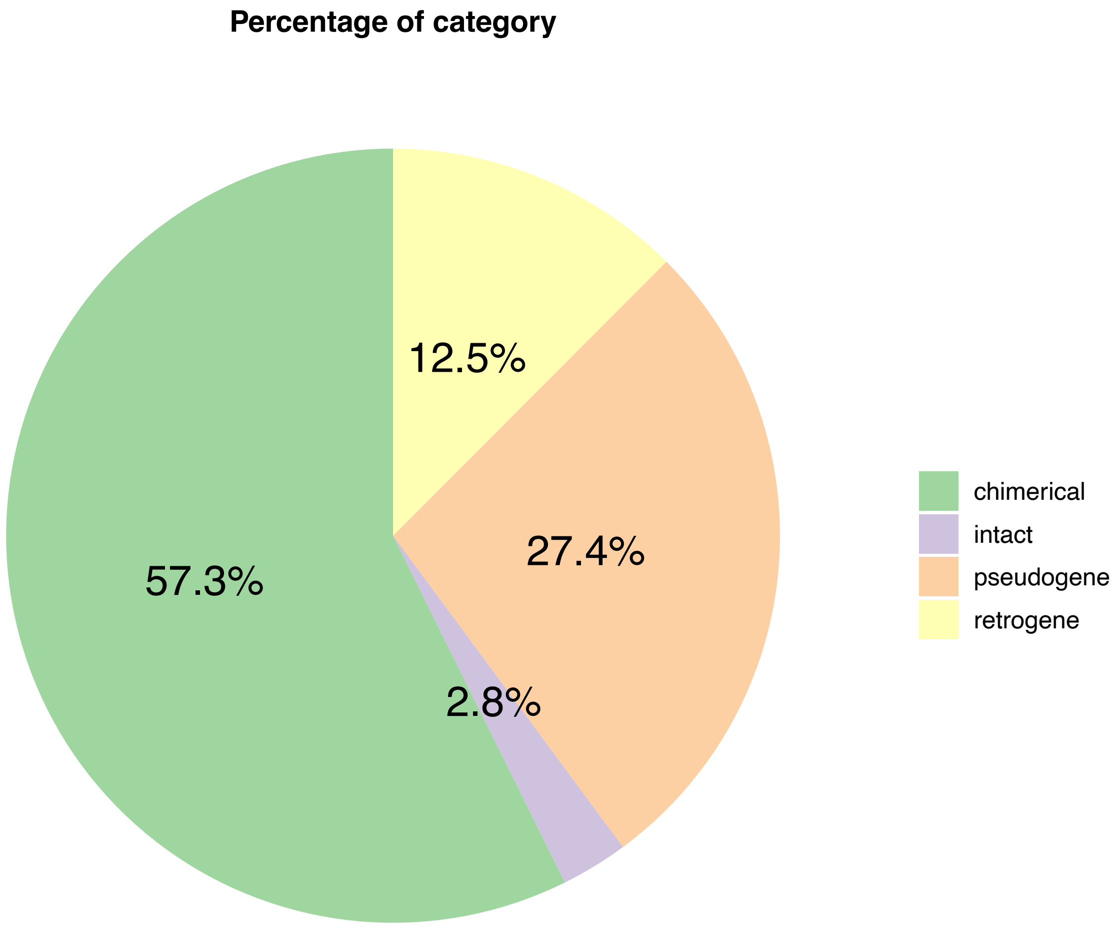

Sup Figure S10. Percentage of different classes of retrogenes present in *P. cineraria*.

A) Leaf transcriptome PCA

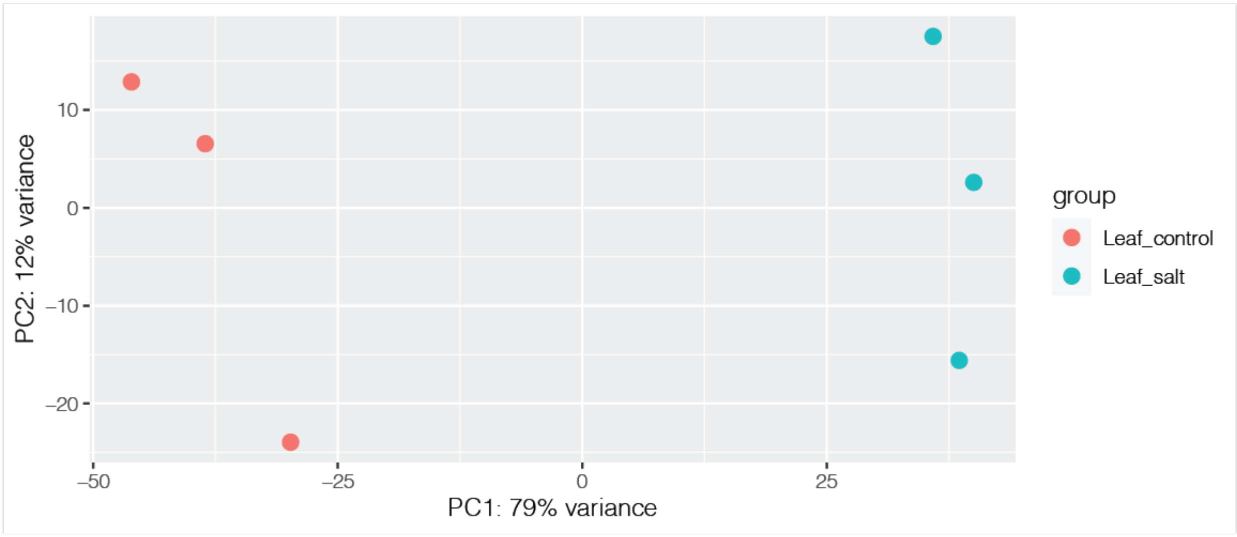

B) Root transcriptome PCA

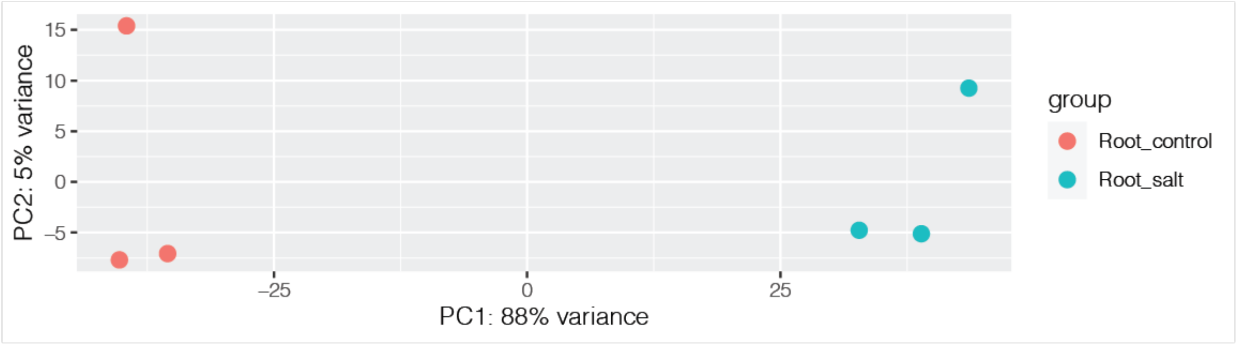

Sup Figure S11. PCA plot A) Leaf transcriptome PCA and B) Root transcriptome PCA
