# Supplementary material for: The Genome of the Mimosoid Legume Prosopis cineraria, a Desert Tree
Source: Int J Mol Sci. 2022 Jul 31;23(15):8503. doi: 10.3390/ijms23158503 (PMC9369113; doi:10.3390/ijms23158503)
Supplement: Supplementary file 1 [file ijms-23-08503-s001.zip › PC_Supplementary_information_final_26_07_22.pdf]

Title: The genome of the mimosoid legume *Prosopis cineraria*, a desert tree

Naganeeswaran Sudalaimuthuasari[1], Rashid Ali[1,2], Martin Kottackal[1], Mohammed Rafi[1], Mariam Al Nuaimi[1], Biduth Kundu[3], Raja Saeed Al-Maskari[3], Xuewen Wang[4], Ajay Kumar Mishra[1], Jithin Balan[1], Srinivasa R. Chaluvadi[4], Fatima Al Ansari[3], Jeffrey L. Bennetzen[4], Michael D. Purugganan[5,6], Khaled M. Hazzouri[1]*, Khaled M.A. Amiri[1,3]*

[1]Khalifa Center for Genetic Engineering and Biotechnology, United Arab Emirates University, P.O. Box. Al Ain 15551, UAE

[2]Mitrix Bio., 400 Farmington Ave., Farmington, Connecticut, CT 06032, USA

[3]Department of Biology, College of Science, United Arab Emirates University, P.O. Box. Al Ain 15551, UAE

[4]Department of Genetics, University of Georgia, Athens, GA 30602, USA

[5]Center for Genomics and Systems Biology, New York University Abu Dhabi, Abu Dhabi 129188, UAE

[6]Center for Genomics and Systems Biology, New York University, New York, NY 10003, USA

A. Genomic DNA isolation method

The leaf tissues collected and stored at -80 °C were ground in liquid nitrogen and 200-300 mg tissue powder was used for genomic DNA extraction. The ground tissues were transferred to 5 ml of extraction buffer [0.1 M Tris pH 7.5, 1 M NaCl, 0.01 M EDTA, 1 % (w/v) CTAB, 2 % (w/v) Polyvinylpyrrolidone and 5 % (v/v) ß-Mercaptoethanol] in 15 ml Falcon tube. ß-Mercaptoethanol was added to the buffer just before use. The tissue powder in buffer was incubated at 65 °C for 30 min with occasional gentle mixing. An equal volume of Chloroform: Isoamyl alcohol (CIA, 24:1) was added and the tubes were incubated at room temperature (RT) with gentle shaking for 10 min. The samples were centrifuged at 12,000 g for 10 min and the supernatant was carefully transferred into another tube. The supernatant was extracted again with an equal volume of CIA and the aqueous phase was precipitated with an equal volume of absolute ethanol. The DNA was precipitated by centrifuging at 12,000 g for 10 min. The pellet was dissolved in 500 µl high salt TE with 2% SDS and added an equal volume of CIA, mixed well by inversion incubated at RT for 10 min with gentle shaking. The aqueous phase after centrifugation at 12000 g for 10 min was transferred into a microcentrifuge tube and was added with 10 µl RNase and mixed well by inversion. After incubation at 37 oC for 10 min, an equal volume of CIA was added and mix well by inversion followed by centrifugation at 12000 g for 10 min. The DNA was precipitated from the aqueous phase by adding an equal volume of absolute ethanol by centrifuging at 12000 g 10 min. The precipitated DNA was washed twice with 70 % ethanol, air-dried, and dissolved in 100 µl of 10 mM Tris buffer (pH 8.0).

B. RNA isolation method

Samples were first homogenized to a fine powder in liquid nitrogen using a pre-chilled mortar and pestle. Approximately 100-200 mg of homogenized powder was added to 1ml 65°C extraction buffer with 2% beta-mercaptoethanol (freshly added to the buffer), in 2ml Eppendorf centrifuge tubes. Tubes were incubated for 10 min at 65°C. The lysate was centrifuged for 10 min at 14,000 rpm to remove cell debris. The clear supernatant was transferred to a clean 2 ml centrifuge tube and extracted twice with an equal volume of chloroform/isoamyl alcohol (24:1). The final aqueous phase was mixed with ¼ volume of 10M LiCl, then mixed and incubated overnight at -20°C to precipitate RNA. Precipitated RNA was harvested by centrifugation at 40,000 rpm for 30 min at 4°C. The RNA pellet was washed 3X with 70% ethanol, dried and resuspended in DNase- and RNase-free water.

C. Protein models used for gene prediction

Plants	ID (NCBI)	Number of proteins
Arabidopsis thaliana	GCF_000001735	48265
Vitis vinifera	GCF_000003745	41208
Glycine max	GCF_000004515	74248
Solanum lycopersicum	GCF_000188115	37658
Theobroma cacao	GCF_000208745	30854
Cicer arietinum	GCF_000331145	35679
Cajanus cajan	GCF_000340665	41387
Vigna radiata	GCF_000741045	42284
Vigna angularis	GCF_001190045	37769
Vigna unguiculata	GCF_004118075	41173
Prosopis alba	GCF_004799145	57572
	**Total**	**488097**

D. Transcriptome data generated for training gene models

Sample_name	Tissue	Number of PE reads
DT_Root_01	Root	35323736
DT_Root_02	Root	37627240
DT_Root_03	Root	35043625
DT_Shoot_01	Shoot	25035375
DT_Shoot_02	Shoot	36270077
DT_Shoot_03	Shoot	31021941
Flower_01_1	Flower	29141695
Flower_01_1	Flower	25589007

E. Salt stress transcriptome study design

	Control	Salt stress (250 mM NaCl, 48 hours)
Root	3 replicates	3 replicates
Shoot	3 replicates	3 replicates

F. Salt stress data and alignment statistics

Sample name	Sample	Number of raw reads	Number of reads after trimming	Alignment %
RC01	Root_Control_1	53216428	49978259	89.7
RC02	Root_Control_2	51942759	49068866	88.9
RC03	Root_Control_3	54608432	51248243	91.0
RN01	Root_Salt_1	62645920	59252232	76.0
RN02	Root_Salt_2	59577573	56335495	81.1
RN03	Root_Salt_3	51093058	48367047	83.3
SC01	Shoot_Control_1	51382394	48383123	96.3
SC02	Shoot_Control_2	51386651	48349343	92.5
SC03	Shoot_Control_3	57856605	54477161	96.4
SN01	Shoot_Salt_1	63338813	59725003	93.8
SN02	Shoot_Salt_2	54074658	51299639	92.4
SN03	Shoot_Salt_3	62069539	58743416	95.6
	**Total**	**673192830**	**635227827**	**94.3**
