# Supplementary material for: The Genome of the Mimosoid Legume Prosopis cineraria, a Desert Tree
Source: Int J Mol Sci. 2022 Jul 31;23(15):8503. doi: 10.3390/ijms23158503 (PMC9369113; doi:10.3390/ijms23158503)
Supplement: Supplementary file 1 [file ijms-23-08503-s001.zip › Sup_Table_S1.pdf]

Supplementary Table S1. Raw sequencing data generated for whole genome sequencing of wild *Prosopis cineraria*.

	Sequencing technology	Number of reads	Coverage*
Short-gun reads	Illumina	~997 million reads	213X
Long reads	Pacio	~7.7 million reads	65X
Long insert Omini-C data	Dovetail-Illumina	~306 million reads	60X

*Coverage calculated by considering the genome size 700 Mb.
