# Supplementary material for: The Genome of the Mimosoid Legume Prosopis cineraria, a Desert Tree
Source: Int J Mol Sci. 2022 Jul 31;23(15):8503. doi: 10.3390/ijms23158503 (PMC9369113; doi:10.3390/ijms23158503)
Supplement: Supplementary file 1 [file ijms-23-08503-s001.zip › Sup_Table_S2.pdf]

Supplementary Table S2. *P. cineraria* genome assembly statistics

	**Contigs**	**Scaffolds (Psudo_chromosome)**
Total_sequences:	3940	2271
Total_bases:	691688661	691857940
################Nucleotide Distribution################		
A_percentage:	33.94308411	33.95953756
T_percentage:	33.96328092	33.93021073
G_percentage:	16.04958506	16.04534885
C_percentage:	16.04404991	16.04043252
(A+T)_percentage:	67.90636503	67.88974829
(G+C)_percentage:	32.09363497	32.08578137
N_percentage:	0	0.024470341
###		
Min_sequence_length:	12499	4999
Max_sequence_length:	9374972	59799197
Average_sequence_length:	175555.5	304649.03
Median_sequence_length:	47023.5	35734
N25_length:	1704465	46246896
L25_number	58	4
N50_length:	649960	41482946
L50_number	234	8
N75_length:	200407	35466890
L75_number	703	12
N90_length:	49164	56968
L90_number	1880	291
N95_length:	35473	37109
L95_number	2709	1058
###		
Sequence_length 1001 - 3000	0	0
Sequence_length 3001 - 5000	0	8
Sequence_length 5001 - 7000	0	0
Sequence_length 7001 - 10k	0	0
Sequence_length 10001 - .1MB	2895	2220
Sequence_length 100001 - 1MB	923	28
Sequence_length > 1MB	122	15

	C:98.9%[S:83.1%,D:15.8%],F:0.2%,M:0.9%,n:425	C:98.8%[S:84.0%,D:14.8%],F:0.2%,M:1.0%,n:425
Complete BUSCOs (C)	420	420
Complete and single-copy BUSCOs (S)	353	357
Complete and duplicated BUSCOs (D)	67	63
Fragmented BUSCOs (F)	1	1
Missing BUSCOs (M)	4	4
Total BUSCO groups searched	425	425
