# Supplementary material for: The Genome of the Mimosoid Legume Prosopis cineraria, a Desert Tree
Source: Int J Mol Sci. 2022 Jul 31;23(15):8503. doi: 10.3390/ijms23158503 (PMC9369113; doi:10.3390/ijms23158503)
Supplement: Supplementary file 1 [file ijms-23-08503-s001.zip › Sup_Table_S3.pdf]

Supplementary Table S3. Top assembled scaffold size distribution

Scaffold ID	Size (Bp)
PC1	59,799,197
PC2	48,612,936
PC3	48,361,062
PC4	43,471,052
PC5	41,482,946
PC6	43,567,763
PC7	39,350,235
PC8	35,466,890
PC9	33,269,108
PC10	39,396,545
PC11	46,246,896
PC12	44,025,251
PC13	40,365,541
PC14	31,271,825
*Others	97,170,693

*Others: Remaining all scaffolds
